# Pathway analysis of peripheral blood CD8+ T cell transcriptome shows differential regulation of sphingolipid signaling in multiple sclerosis and glioblastoma

**DOI:** 10.1371/journal.pone.0305042

**Published:** 2024-06-11

**Authors:** Milan Stefanović, Ivan Jovanović, Maja Živković, Aleksandra Stanković

**Affiliations:** VINČA Institute of Nuclear Sciences—National Institute of the Republic of Serbia, Laboratory for Radiobiology and Molecular Genetics, University of Belgrade, Belgrade, Serbia; Royal Adelaide Hospital, AUSTRALIA

## Abstract

Multiple sclerosis (MS) and glioblastoma (GBM) are CNS diseases in whose development and progression immune privilege is intimately important, but in a relatively opposite manner. Maintenance and strengthening of immune privilege have been shown to be an important mechanism in glioblastoma immune evasion, while the breakdown of immune privilege leads to MS initiation and exacerbation. We hypothesize that molecular signaling pathways can be oppositely regulated in peripheral blood CD8+ T cells of MS and glioblastoma patients at a transcriptional level. We analyzed publicly available data of the peripheral blood CD8+ T cell MS *vs*. control (MSvsCTRL) and GBM *vs*. control (GBMvsCTRL) differentially expressed gene (DEG) contrasts with Qiagen’s Ingenuity pathway analysis software (IPA). We have identified sphingolipid signaling pathway which was significantly downregulated in the GBMvsCTRL and upregulated in the MSvsCTRL. As the pathway is important for the CD8+ T lymphocytes CNS infiltration, this result is in line with our previously stated hypothesis. Comparing publicly available lists of differentially expressed serum exosomal miRNAs from MSvsCTRL and GBMvsCTRL contrasts, we have identified that hsa-miR-182-5p has the greatest potential effect on sphingolipid signaling regarding the number of regulated DEGs in the GBMvsCTRL contrast, while not being able to find any relevant potential sphingolipid signaling target transcripts in the MSvsCTRL contrast. We conclude that the sphingolipid signaling pathway is a top oppositely regulated pathway in peripheral blood CD8+ T cells from GBM and MS, and might be crucial for the differences in CNS immune privilege maintenance of investigated diseases, but further experimental research is necessary.

## 1. Introduction

The blood–brain barrier (BBB) is a physiological barrier formed by the endothelial cells (ECs) of the blood vessel walls to maintain a stable environment for the distribution of resident immune cells (perivascular macrophages and microglial cells) in the central nervous system (CNS) and circulation [1]. The BBB is important to provide immune privilege for the CNS and reduction of immune mediated CNS damage [2]. Immune privilege is the feature of adaptive immune system of avoiding lymphocyte migration through specific tissues, greatly reducing the probability and level of potential immune cell infiltration and inflammation [3].

Glioblastoma (GBM) and multiple sclerosis (MS) are two CNS diseases which have a relatively opposite effect on the maintenance of CNS immune privilege [4]. MS is a chronic autoimmune disease of the CNS, the most common cause of neurological disability in the young adult population (aged 18–40 years) and is still incurable, despite advancements in immunomodulation therapies [5]. Its pathogenesis is defined by CNS immune privilege breakdown and immune cell activation and transmigration in the CNS [6]. CD8+ T lymphocytes mediate focal destruction of myelin sheath and underlying nerve fibers in autoimmune attacks [7]. Self-reactive lymphocytes found in CNS lesions, mainly CD4+ T helper cells, are known to release inflammatory cytokine and chemokines and have been shown to activate resident astrocytes and microglia, increase antigen-presenting cells and effector lymphocyte activity leading to neuroinflammation, disruption of BBB and neurodegeneration [8]. The presence of myelin basic protein and myelin oligodendrocyte glycoprotein antigens activate immune cells in the CNS, leading to chronic demyelinating and autoimmune processes in the CNS [9]. Other theories suggest the role of B cells in immunopathogenesis of MS [10].

GBM is the most common aggressive tumor of the CNS in adults with a poor prognosis in spite of maximal therapy including surgical resection, radiation, and chemotherapy [11]. Infiltration and proliferation of CD8+ T lymphocytes in GBM is associated with improved survival in GBM patients [12], while CD8+ CNS infiltration is associated with active lesions and demyelination in MS patients which leads to progression of the disease and disability [7]. Contrary to MS, GBM exhibits profound local and systemic suppression of adaptive immune response and maintenance of immune privilege, limiting the efficacy of therapeutic strategies and patient survival through immunotherapy [13]. Recently, it was demonstrated that extracellular vesicles (EVs) produced by GBM could change surrounding tumor cells’ phenotype to be more aggressive and have a role in tumor escape from immunosurveillance [14]. EVs, which are considered cellular crosstalk transporters of information, are produced by most known cell types in healthy donors and patients with various pathologies [15]. In immune response modulation, EVs have an important role in the bidirectional communication between the CNS and periphery, and therefore, can be considered as easily accessible transporters of therapies [16]. EVs released from the CNS are involved in the pathogenesis of MS [17], but can also contribute to the repair of demyelinating lesions [18]. The understanding of immune privilege is essential for the development of new therapies for different CNS diseases. It is of importance to understand immunosuppression especially in GBM [19] and relapsing and progressive MS [20] to reveal new therapeutic avenues to treat these two major diseases of the CNS. Groundbreaking discovery of small non-coding RNAs–micro RNAs as EV cargo, which can alter expression of messenger RNA (mRNA) in the CNS and periphery, revealed EV miRNAs as new therapeutic targets [21]. MiRNAs influence GBM growth and progression [22] and MS progression and severity [23].

Therefore, to understand differences in molecular mechanisms pertaining to CNS immune privilege in GBM and MS, we aim to perform comparative transcriptomic analysis of peripheral blood CD8+ T cells from GBM and relapsing-remitting MS (RRMS) patients by analyzing the differentially expressed mRNA genes (DEGs) relative to controls (CTRL), from online sources [24, 25]. To analyze if EV miRNA could through specific miRNA-mRNA interactions influence observed mRNA expression patterns in relevant molecular signaling pathways observed in CD8+ T cells, we aim to compare previously reported serum exosomal miRNAs differentially expressed (DEmiRNAs) in RRMS and GBM patients relative to controls [26, 27] with differentially expressed mRNAs to identify potential mRNA targets.

Our comparative transcriptome analysis has identified the sphingolipid signaling pathway as the top oppositely regulated pathway in CD8+ T cells in GBM and MS, which might be a molecular process relevant to the explanation of differences in immune privilege in two CNS targeting diseases. We have also identified hsa-miR-182-5p as the differentially expressed serum exosomal miRNA which is the most likely to contribute to sphingolipid signaling downregulation in CD8+ T cells of GBM patients.

## 2. Materials and methods

### 2.1. Obtaining publicly available data

Data used for statistical re-analysis of DEGs was obtained from publicly available sources. FASTQ files containing mRNA-mapping reads (Illumina NovaSeq 6000) from CD8+ T cells from GBM patients (n = 8) and their age matched healthy CTRL (n = 6) were downloaded from GEO database entry GSE171197 (downloaded on 03.15.2023.). The list of DEGs between peripheral blood CD8+ T cells of RRMS patients and healthy controls was obtained from Brorson et al., 2020 [24]. Lists of DEmiRNAs from RRMS *vs*. CTRL and GBM *vs*. CTRL contrasts were obtained from Ebrahimkhani et al., 2017 [26] and Ebrahimkhani et al., 2018 [27] respectively. Used publicly available data did not contain information of patients and individual participants could not be identified from the obtained data. None of the GBM and MS patients involved in the study of DEGs in peripheral blood CD8+ T cells were receiving disease treatment at the moment of blood collection [24, 25]. Six out of 14 RRMS patients who donated blood for serum exosomal miRNA were on MS treatment [26], while 3 out of 12 GBM patients whose serum exosomal miRNAs were sequenced have been receiving GBM prescription treatment [27].

### 2.2. Differential mRNA expression analysis from FASTQ files

FASTQ files from GSE171197 entry were analyzed for differential expression using the online bioinformatics platform Galaxy (https://usegalaxy.org). Quality of the raw RNASeq reads was analyzed with the FastQC tool. After the FASTQ files have passed the quality test, RNA sequencing adapters were excluded from the reads sequences using the Cutadapt tool. Processed reads were mapped to the reference genome ((Homo sapiens) (b38); hg38) using the RNA STAR tool. Mapping quality of the BAM files was assessed with the RNA STAR tool, while counting of mapped reads was performed using the featureCounts tool. Graphical presentation of the data and identification of DEGs was performed for the defined contrast using limma voom package. Differential expression of the genes was defined at an adjusted p-value < 0.05 (Benjamini and Hochberg’s, BH, method of adjustment for multiple testing was used) and presented as absolute logarithmic fold change (log2FC).

### 2.3. Bioinformatic analysis

Pathway analysis of both DEG lists in CD8+ T cells (from GBMvsCTRL and MSvsCTRL contrasts was performed using Qiagen’s Ingenuity pathway analysis (IPA, https://digitalinsights.qiagen.com/IPA, spring 2023 release) [28] based on the Ingenuity canonical pathways module with a DEG inclusion criteria of p adj. < 0.05.

To identify oppositely regulated individual DEGs from the GBMvsCTRL and MSvsCTRL contrasts, we have performed an intersection of the DEG lists and retained those which were oppositely regulated between GBM and MS CD8+ T cells relative to controls. DEGs passing previous filtering method were subsequently annotated in the Kyoto Encyclopedia of Genes and Genomes (KEGG) to identify their involvement in the respective pathways.

For the purpose of finding DEmiRNAs which have the potential to target pathway-specific DEGs, we have initially generated lists of reported and predicted interacting miRNAs for all gene members of enriched molecular pathways using gene2mir tool of the miRNet software (https://www.mirnet.ca, last accessed in April 2023). Subsequently, only interactions relevant to previously identified DEGs have been retained. The extracted interactions were used to perform the intersection with lists of serum exosomal DEmiRNAs to prioritize miRNAs with a potentially regulatory role on DEGs which are members of the corresponding pathways. Finally, we have compared the direction of differential expression of DEmiRNAs with the direction of differential expression in DEGs to further analyze the possibility of individual miRNAs affecting the differential expression of mRNAs in molecular pathways in a canonical manner (downregulation of gene expression).

## 3. Results

### 3.1. GBMvsCTRL differential gene expression

**Fig 1:**
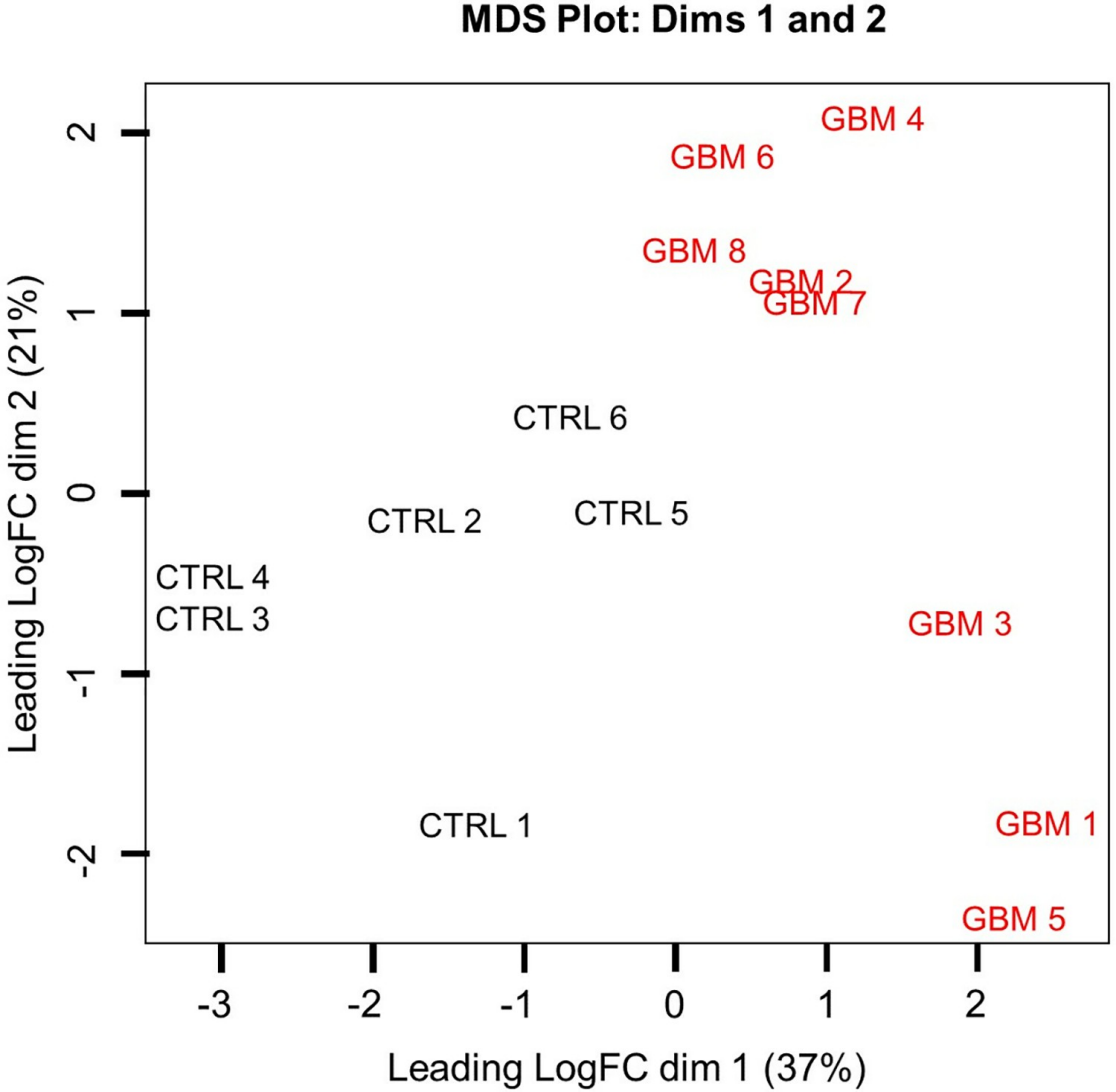
Principal component analysis plot for leading Log2FC genes in peripheral blood CD8+ T cell transcriptome of GBM patients and healthy CTRL samples. Red lettered entries represent GBM patient samples, while black lettered entries represent CTRL samples. Samples are annotated with their GEO accession numbers in S1 Table. GBM–Glioblastoma patients; CTRL–Control samples.

Analysis of the data obtained from the GSE171197 transcriptome profiling dataset led to the identification of 3852 differentially expressed mRNAs in peripheral blood CD8+ T lymphocytes between GBM patients and age-matched healthy CTRL, of which 2004 were upregulated while 1848 genes were downregulated in GBM patients (adjusted p-value < 0.05) (S1 Table). Principal component analysis has demonstrated a clear split between the analyzed groups (Fig 1) supporting the identification of a relatively high number of DEGs and warranting further bioinformatic interpretation regarding molecular pathway enrichment.

### 3.2. Sphingolipid signaling pathway is oppositely-regulated between MSvsCTRL and GBMvsCTRL DEGs from peripheral blood CD8+ T lymphocytes

**Fig 2:**
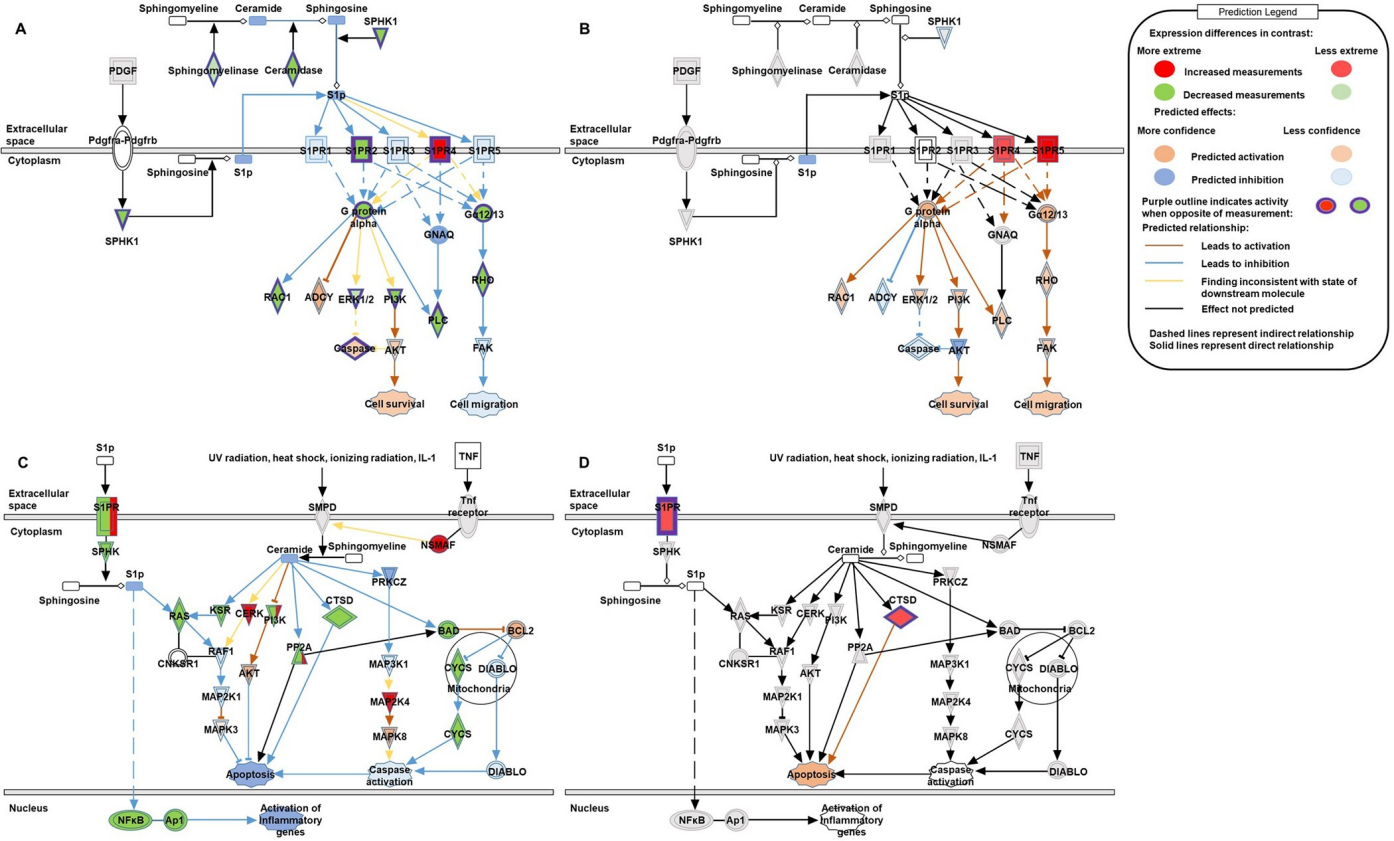
Sphingolipid signaling pathway illustrations presenting effects of differential gene expressions. (A) Sphingosine-1-phosphate signaling in the GBMvsCTRL contrast. (B) Sphingosine-1-phosphate signaling in the MSvsCTRL contrast. (C) Ceramide signaling in the GBMvsCTRL contrast. (D) Ceramide signaling in the MSvsCTRL contrast. Effects on downstream molecules and cellular functions are predicted and calculated with Qiagen’s Ingenuity Pathway Analysis algorithm. GBM–glioblastoma patients; CTRL–healthy controls; MS–multiple sclerosis patients.

To further examine the biological meaning of the transcriptional profiles from the investigated contrasts, we analyzed the two lists of DEGs (MSvsCTRL and GBMvsCTRL) using the Canonical pathway module of the IPA tool. Full list of IPA Canonical pathways enriched in DEGs of GBMvsCTRL and MSvsCTRL contrasts is presented in S2 Table. For the investigated contrasts (GBMvsCTRL and MSvsCTRL) ceramide signaling, sphingosine-1-phosphate signaling, IL-33 signaling pathway, 2-oxobutanoate degradation I, superpathway of methionine degradation, BMP signaling pathway, sumoylation pathway and adipogenesis pathway have been identified as commonly significantly enriched pathways in both lists of DEGs. Ceramide signaling was the top of the common enriched pathways in both investigated contrasts (Table 1). Sphingosine-1-phosphate signaling pathway was also identified as commonly enriched. Along with ceramide signaling, these two pathways constitute the sphingolipid signaling pathway. Ceramide signaling had 33 DEGs from the GBMvsCTRL contrast (22 downregulated and 11 upregulated) and 3 DEGs from the MSvsCTRL contrast (CTSD, S1PR4 and S1PR5, all upregulated) while Sphingosine-1-phosphate signaling contained 30 DEGs from the GBMvsCTRL contrast (21 downregulated and 9 upregulated) and 2 DEGs from the MSvsCTRL contrast (S1PR4 and S1PR5, both upregulated). The negative Z score in GBMvsCTRL shows the cumulative gene expression effect on a both sphingosine-1-phosphate and ceramide signaling pathway depicting significant downregulation in signaling. It should be noted that Z score signaling could not be calculated for MSvsCTRL contrast in the case of the described pathways. However, all of the DEGs from MSvsCTRL contrast involved in these pathways were upregulated and identified to have a positive effect on corresponding signaling pathways according to IPA. This suggest the potential upregulation of the both, sphingosine-1-phosphate and ceramide signaling pathways. DEG interactions with downstream molecules and cellular functions in the sphingolipid pathway, as predicted and calculated by IPA, are illustrated in Fig 2. Effects of DEGs in the sphingolipid signaling pathway are shown in S2 Table.

**Table 1:** Mutually enriched IPA Canonical pathways that belong to sphingolipid signaling in GBMvsCTRL and MSvsCTRL contrasts.

IPA Canonical pathway	GBMvsCTRL			MSvsCTRL		
	p adj,	Ratio	Z score	p adj.	Ratio	Z score
Ceramide Signaling	1x10^-6^	33/91	-2.117	4x10^-4^	3/91	NA
Sphingosine-1-phosphate Signaling	0.006	30/120	-2.353	0.014	2/120	NA

IPA–Qiagen Ingenuity Pathway Analysis; GBMvsCTRL–DEGs between CD8+ T cells from peripheral blood of GBM patients vs. age-matched healthy CTRL; MSvsCTRL–DEGs between CD8+ T cells from peripheral blood of RRMS patients vs. age-matched healthy CTRL; GBM–glioblastoma patients; RRMS–relapsing-remitting multiple sclerosis; CTRL–healthy controls; DEG–differentially expressed mRNA genes; p adj.–adjusted p value of < 0.05 is considered as statistically significant pathway enriched in DEGs; Ratio–number of DEGs in a pathway/all genes in that pathway; Z-score–describes the cumulative gene expression effect on pathway signaling; NA–IPA was unable to calculate Z-score for the contrast DEGs.

### 3.3. DEGs oppositely-regulated between GBMvsCTRL and MSvsCTRL contrasts

We have additionally performed the analysis of the oppositely regulated DEGs between GBMvsCTRL and MSvsCTRL contrasts and characterized their pathway involvement using the KEGG database. Five DEGs were oppositely-regulated between MSvsCTRL and GBMvsCTRL DEG lists, of which all were upregulated in MS patients and downregulated in GBM patients compared to CTRL. Of the identified oppositely regulated intersecting genes, *CTSD* was found to be involved in sphingolipid signaling pathway (Table 2).

**Table 2:** Five oppositely-regulated differentially expressed genes between GBMvsCTRL and MSvsCTRL contrasts.

GENE	MSvsCTRL Log2FC	MSvsCTRL p adj.	GBMvsCTRL Log2FC	GBMvsCTRL p adj.	KEGG pathway
*H2AX*	0.724	0.021	-1.4451	0.003	• hsa03082 ATP-dependent chromatin remodeling—Homo sapiens (human) • hsa04217 Necroptosis—Homo sapiens (human) • hsa04613 Neutrophil extracellular trap formation—Homo sapiens (human) • hsa05034 Alcoholism—Homo sapiens (human) • hsa05322 Systemic lupus erythematosus—Homo sapiens (human)
*JUND*	0.510	0.036	-3.70787	8x10^-6^	• hsa04010 MAPK signaling pathway—Homo sapiens (human) • hsa04380 Osteoclast differentiation—Homo sapiens (human) • hsa04657 IL-17 signaling pathway—Homo sapiens (human); • hsa04928 Parathyroid hormone synthesis, secretion and action—Homo sapiens (human)
*MIDN*	0.447	0.036	-3.51161	7x10^-6^	NA
*RBM38*	0.372	0.037	-2.76309	4.5x10^-6^	NA
*CTSD*	0.280	0.039	-1.14923	0.013	• hsa04071 Sphingolipid signaling pathway—Homo sapiens (human) • hsa04140 Autophagy—animal—Homo sapiens (human) • hsa04142 Lysosome—Homo sapiens (human) • hsa04210 Apoptosis—Homo sapiens (human) • hsa04915 Estrogen signaling pathway—Homo sapiens (human) • hsa05152 Tuberculosis—Homo sapiens (human) • hsa05415 Diabetic cardiomyopathy—Homo sapiens (human)

GBMvsCTRL–DEGs between CD8+ T cells from peripheral blood of GBM patients vs. age-matched healthy CTRL; DEGs between MSvsCTRL–CD8+ T cells from peripheral blood of RRMS patients vs. age-matched healthy CTRL; GBM–glioblastoma patients; RRMS–relapsing-remitting multiple sclerosis; CTRL–healthy controls; DEG–differentially expressed mRNA genes; KEGG—Kyoto Encyclopedia of Genes and Genomes; p adj.–adjusted p value of < 0.05 is considered statistically significant pathway enriched in DEGs; Log2FC–logarithm base 2 of fold change; NA–not a member of a defined KEGG pathway.

### 3.4. Serum DEmiRNAs in glioblastoma have potential targets in the sphingolipid signaling DEGs

By analyzing the interactions of the all known sphingolipid pathway genes and their miRNAs, a list of miRNA-gene interactions has been generated. From the conceived list, DEmiRNAs interacting with GBMvsCTRL and MSvsCTRL DEGs were filtered. We have discovered that 40 sphingolipid pathway DEGs in the GBMvsCTRL contrast were potential DEmiRNA targets (shown in S2 Table). Contrary there were no exosomal serum DEmiRNAs targeting MSvsCTRL DEGs. We have also analyzed the potential miRNA- mRNA interactions in the context of the direction of differential expressions of GBMvsCTRL miRNAs and mRNAs. By following the canonical miRNA activity stating that miRNA-mRNA interaction leads to mRNA degradation, we have identified hsa-miR-182-5p as a serum exosomal DEmiRNA with the greatest potential effect on the observed downregulation on sphingolipid signaling DEGs in peripheral blood CD8+ T cells of GBM patients (19 hits, 16 of which are downregulated). The DEmiRNA list with proportions of target DEGs with expected directions of differential expression regarding miRNA activity is shown in Table 3.

**Table 3:** MSvsCTRL and GBMvsCTRL exosomal serum DEmiRNAs target DEGs in sphingolipid signaling and their expression regarding canonical miRNA activity.

Serum exosomal DEmiRNA	Direction of differential expression	Contrast	Expected regulation/total number of DEG targets in sphingolipid signaling
hsa-miR-486-5p	UP	GBMvsCTRL	2/2
hsa-miR-182-5p	UP	GBMvsCTRL	16/19
hsa-miR-486-3p	UP	GBMvsCTRL	0/0
hsa-miR-183-5p	UP	GBMvsCTRL	8/10
hsa-miR-378a-3p	UP	GBMvsCTRL	9/11
hsa-miR-501-3p	UP	GBMvsCTRL	1/1
hsa-miR-20b-5p	UP	GBMvsCTRL	8/13
hsa-miR-106b-3p	UP	GBMvsCTRL	1/1
hsa-miR-629-5p	UP	GBMvsCTRL	0/0
hsa-miR-185-5p	UP	GBMvsCTRL	6/7
hsa-miR-25-3p	UP	GBMvsCTRL	5/11
hsa-miR-7d-3p	DOWN	GBMvsCTRL	2/9
hsa-miR-21-5p	DOWN	GBMvsCTRL	4/11
hsa-miR-409-3p	DOWN	GBMvsCTRL	0/0
hsa-miR-381-3p	DOWN	GBMvsCTRL	0/2
hsa-miR-7a-3p	DOWN	GBMvsCTRL	1/2
hsa-miR-130b-5p	DOWN	GBMvsCTRL	0/4
hsa-miR-328-3p	DOWN	GBMvsCTRL	0/1
hsa-miR-323b-3p	DOWN	GBMvsCTRL	0/1
hsa-miR-126-5p	DOWN	GBMvsCTRL	3/6
hsa-miR-493-5p	DOWN	GBMvsCTRL	0/4
hsa-miR-340-5p	DOWN	GBMvsCTRL	2/6
hsa-miR-339-5p	DOWN	GBMvsCTRL	1/5
hsa-miR-654-3p	DOWN	GBMvsCTRL	1/2
hsa-miR-543	DOWN	GBMvsCTRL	0/2
hsa-miR-485-3p	DOWN	GBMvsCTRL	0/2
hsa-miR-15b-5p	UP	MSvsCTRL	0/0
hsa-miR-30b-5p	UP	MSvsCTRL	0/0
hsa-miR-342-3p	UP	MSvsCTRL	0/0
hsa-miR-451a	UP	MSvsCTRL	0/0

GBMvsCTRL–DEmiRNA between serum exosomes of GBM patients vs. age-matched CTRL; MSvsCTRL–DEmiRNA between serum exosomes of RRMS patients vs. CTRL; GBM–glioblastoma patients; RRMS–relapsing-remitting multiple sclerosis patients; CTRL–healthy controls; DEmiRNA–serum exosomal differentially expressed miRNA; DEG–differentially expressed peripheral blood CD8+ T cell mRNA genes; Log2FC—Logarithm base 2 of fold change; Expected regulation/total number of DEG targets in sphingolipid signaling–number of potential target DEGs in the sphingolipid signaling pathway for defined contrast which follow miRNA canonical activity/number of all potential target DEGs in the sphingolipid signaling pathway for defined contrast. Lists of DEmiRNA were extracted from Ebrahimkhani et al., 2017 [26] and Ebrahimkhani et al., 2018 [27].

## 4. Discussion

In this study, we performed *in silico* multi-omics approach to investigate the differential regulation of molecular pathways in the peripheral blood CD8+ lymphocytes between GBM and MS, aiming for further elucidation of the mechanisms involved in maintenance of CNS immune privilege. Our results have directed to sphingolipid signaling as the top oppositely regulated pathway between glioblastoma *vs*. control and MS *vs*. control contrasts.

Sphingolipids are cell membrane lipids, involved in cell proliferation, signaling cascades and apoptosis and the dysregulation of sphingolipid signaling was recognized in neurodegenerative diseases, including MS [29].

Sphingosine-1-phosphate and ceramide signaling canonical pathways constitute the sphingolipid signaling pathway, being separated by the IPA software using their main cellular effects (cell survival and migration for sphingosine-1-phosphate, and apoptosis and inflammatory gene activation for ceramide signaling pathway) [28]. Sphingolipid signaling has been previously shown to play an important role in pathogenic T lymphocyte infiltration in the CNS of humans [30] and rodents [31, 32]. In mouse and rat models of traumatic CNS injury, suppression of sphingosine-1-phosphate signaling leads to reduction of T lymphocyte infiltration, reduced inflammation and reduced BBB disruption [31, 32]. *In vitro* models of BBB have also confirmed the role of sphingosine-1-phosphate signaling in BBB preservation by reducing peripheral blood mononuclear cell transendothelial migration via signaling modulation [33]. In mouse models of encephalomyelitis virus infection, activated CD8+ T cells have been shown to be solely sufficient for BBB disruption [34].

In MS, the sphingolipid pathway could affect CNS immune privilege by: positively influencing effector lymphocyte transmigration from lymphoid tissues to the blood stream and lymphatic system [35, 36]. These mechanisms are most evident with the pharmacophysiological effect of the sphingolipid signaling suppressor drug fingolimod [30, 37]. Fingolimod exhibits negative modulation of sphingosine-1-phosphate signaling by causing the internalization and intracellular degradation of sphingosine-1-phosphate receptors [38–41], and may also negatively regulate the pathway by suppressing sphingosine-1 phosphate production via inhibition of activity and downregulation of expression of sphingosine kinases [37, 42]. In mice and rat model of MS, experimental autoimmune encephalomyelitis (EAE), suppression of sphingosine-1 phosphate signaling with fingolimod leads to lower infiltration of CD8+ and CD4+ T lymphocytes in the CNS, slower disease progression and increased BBB reparation [43–47]. Thus, fingolimod prevents MS-induced BBB disruption [48], effector T cell transendothelial migration [36] and T cell chemotaxis [49]. Therefore, observed potential upregulation of sphingolipid signaling pathways in the peripheral blood CD8+ T cell DEGs in MSvsCTRL contrast is expected due to its essential role in lymphocyte trafficking and entry into inflamed tissues [49, 50]. It is crucial to underlie that according to the original publication [24] none of the included RRMS patients were receiving therapy, thus no influence of treatment on our results could be possible. The ceramide signaling “sub-pathway” of sphingolipid signaling was also shown to be crucial for the infiltration of CD8+ and CD4+ T lymphocytes in EAE, as mice which are knockouts for the acid sphingomyelinase (*Asm*) gene, which produce ceramide, have lower T lymphocyte infiltration into the CNS and better BBB preservation after disease induction [51].

In the glioblastoma tissue, sphingolipid signaling has dual effects on disease exacerbation. Ceramide signaling leads to the apoptosis of tumor cells [52–54] while sphingosine-1-phosphate leads to tumor vascularization, migration and survival of tumor cells [55–57]. However, it has been shown that peripheral blood T lymphocytes have lower Sphingosine-phosphate receptor 1 (S1PR1) expression on cell surface in GBM patients, and mice with intracranial tumors had greater T cell bone marrow sequestration compared to controls [58, 59]. This is in line with our results showing a significant downregulation of the sphingolipid pathways in the peripheral blood CD8+T lymphocytes of GBMvsCTRL contrast, which could be a mechanism of T cell dysfunction caused by cancer, suggested earlier [58, 59]. Although the downregulation of *S1PR1* expression was not statistically significant in GBMvsCTRL contrast in our study, we have identified significant upregulation of *S1PR4* and downregulation of *S1PR2*. The specific function of *S1PR2* receptor in the context of T cell activity is still unclear as its expression does not significantly change in CD8+ T cells during *in vitro* activation [60], and it should be pointed out that *S1PR2* knockout CD4+ T lymphocytes do not exhibit any changes in transendothelial migration [61]. *In vitro* suppression of sphingosine-1-signaling via S1PR2 leads to higher levels of CD4+ and CD8+ effector memory T cells migration, implying that S1PR2 signaling promotes the retention of these lymphocytes in lymphoid organs [62]. Therefore, we cannot suggest that identified *S1PR2* downregulation in peripheral blood CD8+ T cells of GBM patients is a putative mechanism of GBM immune evasion. On the other hand, wild type mice had a lesser abundance of antitumor CD8+ T cells in breast and colon cancer compared to *S1pr4* knockout mice [63], while in an *in vitro* model of nutrient deprivation the stimulation signaling via S1PR4 promotes the expression of immunosuppressive CXCR4 on the cell surface of human CD8+ T cells [64], supporting the identified *S1PR4* upregulation in CD8+ T cells of GBM patients as a potential mechanism of immune evasion.

Regarding the specific DEGs oppositely regulated in the investigated contrasts, we have identified *Cathepsin D* (*CTSD*) as a member of the sphingolipid pathway (upregulated in MSvsCTRL and downregulated in GBMvsCTRL). CTSD is a lysosomal aspartate endoproteinase which promotes the mitochondrial apoptotic pathway via direct proteolytic activation of the proapoptotic Bid protein or indirect destabilization of antiapoptotic Bcl-XL [65–69]. Lysosomal exit and proteolytic activity of CTSD is also promoted by ceramides [67, 70, 71]. This is in line with results describing that peripheral blood CD8+ and CD4+ T cells of RRMS patients are more prone to apoptosis compared to CTRL *ex vivo* [72] and *in vivo* [73, 74]. However, the downregulation of CTSD in the GBMvsCTRL contrast might imply that apoptosis is downregulated in peripheral blood CD8+ T cells of GBM patients, which would be contrary to the documented increase of apoptotic elimination of peripheral blood T lymphocytes in patients with primary intracranial tumors [75], and *in vitro* capacity of GBM cell culture medium to promote CD8+ T cell apoptosis [76]. This suggests a more complex mechanism underlying the opposite regulation of *CTSD* in GBM and MS. However, it should be noted that there was a relatively high discrepancy in the number of identified DEGs between the contrasts. This warrants further studies, and represent the main limitation of the current study, in order to increase the number of samples to precisely discover additional DEGs which could be oppositely regulated and thus providing more key targets of the dysregulated pathways.

One of the factors which may contribute to differential sphingolipid signaling in peripheral blood lymphocytes of MS and GBM patients are differences in the peripheral blood lipidomic profiles of the diseases. Sphingosine-1-phosphate has a higher concentration in plasma of GBM patients compared to controls [77]. While the concentration of sphingosine-1-phosphate is not different between MS patients and healthy controls [78], serum concentration of multiple ceramide species is elevated in MS [79].

In the current study, we further evaluated if differentially expressed exosomal miRNAs might affect expression of the sphingolipid pathway DEGs in investigated phenotypes. We have observed that most of the exosomal serum DEmiRNAs target at least one of the sphingolipid pathway DEGs in GBM. On the other hand, none of the identified MSvsCTRL DEGs in sphingolipid signaling were targets of the RRMS exosomal serum DEmiRNAs. However, it should be noted that only four miRNAs were identified to be differentially expressed in serum exosomes of RRMS patients compared to controls [26] warranting further integrative studies toward future identification of additional exosomal DEmiRNA in MS with potential activity on sphingolipid signaling. Herein, we intentionally included RRMS data set in which age, gender and treatment did not correlate with the expression profiles of the identified DEmiRNAs [26]. Although we did not present evidence of serum exosomal miRNAs affecting sphingolipid signaling in RRMS, in another study the suppression of the pathway via fingolimod therapy led to changes in expression of certain exosomal miRNAs in fingolimod responding RRMS patients [80]. We additionally investigated if the miRNAs presented in Table 3. correspond to suggested fingolimod responsive miRNAs [80] and found no overlap. We also considered the possibility of the effect of GBM treatment on miRNA expression, but it was not addressed in original study as only 25% was on treatment [27]. However, we created a PCA plot using miRNA normalized counts and found that therapy did not differentiate samples from treated glioblastoma patients in comparison to treatment naïve samples.

Cancer derived exosomal cargo including exosomal miRNAs has been suggested to contribute to suppression of antitumor CD8+ T cell activity via their modulation of relevant pathways. For example, CD8+ T cells internalize melanoma-derived exosomes carrying hsa-miR-3187-3p, which contributes to the inhibition of T cell receptor signaling in CD8+ T cells [81]. It has been described that certain GBM derived exosomal cargo can inhibit antitumor cytotoxicity of CD8+ T cells in mice [82] and humans [83]. The hsa-miR-29a, hsa-miR-92a and hsa-miR-1246 from GBM cell derived exosomes indirectly suppress CD8+ T lymphocyte activation by mediating the expansion and activation of myeloid-derived suppressor cells [84, 85]. In the current study we have identified that hsa-miR-182-5p interacts with the largest number of sphingolipid pathway DEGs in GBM, making it the most interesting exosomal miRNA hub of the sphingolipid pathway. In sera of breast cancer patients, the observed upregulation of hsa-miR-182-5p was in line with the downregulation of corresponding target genes in peripheral blood leukocytes, while treatment of Jurkat cells with hsa-miR-182-5p induces their direction to an T regulatory phenotype [86]. The microRNA-183/96/182 cluster expression in lung cells causes IL-2 mediated paracrine activation of effector CD8+ T cell proliferation and function promoting CD8+ T cell antitumor response [87]. In literature, there are inconsistent results regarding the effect of hsa-miR-182-5p on glioblastoma development [88, 89]. Oncogenic receptor *EGFR* amplification is associated with microRNA-183/96/182 cluster upregulation and proposed downregulation of proapoptotic FOXO1 [89] and hypoxic glioblastoma secreted exosomal hsa-miR-182-5p promote cancer survival and angiogenesis [88]. Contrary, hsa-miR-182 expression in GBM is associated with longer patient survival and exogenous miR-182 treatment of mice with intracranial GBM xenografts leads to longer survival and reduced tumor burden [90]. To our knowledge, there is no data associating hsa-miR-182-5p expression in GBM patients with CD8+ T cell function in literature making it an interesting field for future investigation of CD8+ T cell regulation via the miRNA-sphingolipid pathway axis.

Current study offers new insight into sphingolipid signaling as a plausible process of differentiation between the CNS infiltrating CD8+ T lymphocyte with regard to distinct phenotypes and capacity of exosomal cargo to modify it. The understanding of peripheral immune modulation in CNS diseases might open the possibilities to practical and non-invasive approaches in disease management and therapy development. Exosomes are coming of age as a potent carrier of therapeutic agents. Further investigation of miRNA effects on sphingolipid signaling and the mechanisms for precise delivery to target cells may represent a way forward in pursuit of novel avenues in treatment of the two immunologically different but both devastating diseases.

The results of this *in silico* study have underscored the importance of sphingolipid signaling on the peripheral blood CD8+ T lymphocytes transcriptome in MS and GBM, and strongly suggest that sphingolipid signaling might be a mechanism which is behind the differences in CD8+ T cell behavior with regard to CNS infiltration. The results themselves represent a further contribution to the understanding of opposing immunological effects in the two pathophysiologically different CNS diseases by emphasizing the opposite expression of the relevant, and even, crucial immune system molecular pathways with the top cellular players of the immune system in MS and GBM. It adds up to current knowledge of sphingolipids’ role in reciprocal interference and modulation of immune components in immune response. Further link toward regulatory exosomal miRNAs, at least in one of investigated phenotypes, provide insight into possible modulatory interventions relevant for modification of gene expression and enhancement of communication between the periphery and CNS. While envisaging the therapeutic application of EVs in focal CNS diseases, further *in vitro* and *in vivo* experimental validation is necessary to fully understand the importance of sphingolipid signaling in regard to CNS immune privilege.

## Supporting information

S1 Table LimmaVoom mRNA differential expression analysis results. (XLSX)

S2 Table IPA results and Sphingolipid signaling pathway DEGs with potential targeting differentially expressed serum exosomal miRNAs. (XLSX)

## References

[1] Daneman R, Prat A. The blood-brain barrier. Cold Spring Harb Perspect Biol. 2015 Jan 5;7(1):a020412. doi: 10.1101/cshperspect.a020412 25561720 PMC4292164

[2] Proulx ST, Engelhardt B. Central nervous system zoning: How brain barriers establish subdivisions for CNS immune privilege and immune surveillance. J Intern Med. 2022 Jul;292(1):47–67. doi: 10.1111/joim.13469 35184353 PMC9314672

[3] Forrester JV, Xu H, Lambe T, Cornall R. Immune privilege or privileged immunity? Mucosal Immunol. 2008 Sep;1(5):372–81. doi: 10.1038/mi.2008.27 19079201

[4] Xu J, Ma C, Hua M, Li J, Xiang Z, Wu J. CNS and CNS diseases in relation to their immune system. Front Immunol. 2022 Nov 16;13:1063928. doi: 10.3389/fimmu.2022.1063928 36466889 PMC9708890

[5] Thompson AJ, Banwell BL, Barkhof F, Carroll WM, Coetzee T, Comi G, et al. Diagnosis of multiple sclerosis: 2017 revisions of the McDonald criteria. Lancet Neurol. 2018 Feb;17(2):162–173. doi: 10.1016/S1474-4422(17)30470-2 29275977

[6] Nishihara H, Engelhardt B. Brain Barriers and Multiple Sclerosis: Novel Treatment Approaches from a Brain Barriers Perspective. Handb Exp Pharmacol. 2022;273:295–329. doi: 10.1007/164_2020_407 33237504

[7] Machado-Santos J, Saji E, Tröscher AR, Paunovic M, Liblau R, Gabriely G, et al. The compartmentalized inflammatory response in the multiple sclerosis brain is composed of tissue-resident CD8+ T lymphocytes and B cells. Brain. 2018 Jul 1;141(7):2066–2082. doi: 10.1093/brain/awy151 29873694 PMC6022681

[8] Kunkl M, Frascolla S, Amormino C, Volpe E, Tuosto L. T Helper Cells: The Modulators of Inflammation in Multiple Sclerosis. Cells. 2020 Feb 19;9(2):482. doi: 10.3390/cells9020482 32093011 PMC7072830

[9] Egg R, Reindl M, Deisenhammer F, Linington C, Berger T. Anti-MOG and anti-MBP antibody subclasses in multiple sclerosis. Mult Scler. 2001 Oct;7(5):285–9. doi: 10.1177/135245850100700503 11724443

[10] Gharibi T, Babaloo Z, Hosseini A, Marofi F, Ebrahimi-Kalan A, Jahandideh S, et al. The role of B cells in the immunopathogenesis of multiple sclerosis. Immunology. 2020 Aug;160(4):325–335. doi: 10.1111/imm.13198 32249925 PMC7370136

[11] Koshy M, Villano JL, Dolecek TA, Howard A, Mahmood U, Chmura SJ, et al. Improved survival time trends for glioblastoma using the SEER 17 population-based registries. J Neurooncol. 2012 Mar;107(1):207–12. doi: 10.1007/s11060-011-0738-7 21984115 PMC4077033

[12] Mauldin IS, Jo J, Wages NA, Yogendran LV, Mahmutovic A, Young SJ, et al. Proliferating CD8+ T Cell Infiltrates Are Associated with Improved Survival in Glioblastoma. Cells. 2021 Dec 1;10(12):3378. doi: 10.3390/cells10123378 34943886 PMC8699921

[13] Ayasoufi K, Pfaller CK, Evgin L, Khadka RH, Tritz ZP, Goddery EN, et al. Brain cancer induces systemic immunosuppression through release of non-steroid soluble mediators. Brain. 2020 Dec 1;143(12):3629–3652. doi: 10.1093/brain/awaa343 33253355 PMC7954397

[14] Pathania AS, Prathipati P, Challagundla KB. New insights into exosome mediated tumor-immune escape: Clinical perspectives and therapeutic strategies. Biochim Biophys Acta Rev Cancer. 2021 Dec;1876(2):188624. doi: 10.1016/j.bbcan.2021.188624 34487817 PMC8595777

[15] Keller S, Ridinger J, Rupp AK, Janssen JW, Altevogt P. Body fluid derived exosomes as a novel template for clinical diagnostics. J Transl Med. 2011 Jun 8;9:86. doi: 10.1186/1479-5876-9-86 21651777 PMC3118335

[16] Zamboni S, D’Ambrosio A, Margutti P. Extracellular vesicles as contributors in the pathogenesis of multiple sclerosis. Mult Scler Relat Disord. 2023 Mar;71:104554. doi: 10.1016/j.msard.2023.104554 36842311

[17] D’Anca M, Fenoglio C, Buccellato FR, Visconte C, Galimberti D, Scarpini E. Extracellular Vesicles in Multiple Sclerosis: Role in the Pathogenesis and Potential Usefulness as Biomarkers and Therapeutic Tools. Cells. 2021 Jul 8;10(7):1733. doi: 10.3390/cells10071733 34359903 PMC8303489

[18] Dolcetti E, Bruno A, Guadalupi L, Rizzo FR, Musella A, Gentile A, et al. Emerging Role of Extracellular Vesicles in the Pathophysiology of Multiple Sclerosis. Int J Mol Sci. 2020 Oct 4;21(19):7336. doi: 10.3390/ijms21197336 33020408 PMC7582271

[19] Himes BT, Geiger PA, Ayasoufi K, Bhargav AG, Brown DA, Parney IF. Immunosuppression in Glioblastoma: Current Understanding and Therapeutic Implications. Front Oncol. 2021 Oct 28;11:770561. doi: 10.3389/fonc.2021.770561 34778089 PMC8581618

[20] Amin M, Hersh CM. Updates and advances in multiple sclerosis neurotherapeutics. Neurodegener Dis Manag. 2023 Feb;13(1):47–70. doi: 10.2217/nmt-2021-0058 36314777 PMC10072078

[21] Xia X, Wang Y, Huang Y, Zhang H, Lu H, Zheng JC. Exosomal miRNAs in central nervous system diseases: biomarkers, pathological mediators, protective factors and therapeutic agents. Prog Neurobiol. 2019 Dec;183:101694. doi: 10.1016/j.pneurobio.2019.101694 31542363 PMC7323939

[22] Musatova OE, Rubtsov YP. Effects of glioblastoma-derived extracellular vesicles on the functions of immune cells. Front Cell Dev Biol. 2023 Mar 7;11:1060000. doi: 10.3389/fcell.2023.1060000 36960410 PMC10028257

[23] Emami Nejad A, Mostafavi Zadeh SM, Nickho H, Sadoogh Abbasian A, Forouzan A, Ahmadlou M, et al. The role of microRNAs involved in the disorder of blood-brain barrier in the pathogenesis of multiple sclerosis. Front Immunol. 2023 Dec 14;14:1281567. doi: 10.3389/fimmu.2023.1281567 38193092 PMC10773759

[24] Brorson IS, Eriksson AM, Leikfoss IS, Vitelli V, Celius EG, Lüders T, et al. CD8+ T cell gene expression analysis identifies differentially expressed genes between multiple sclerosis patients and healthy controls. Mult Scler J Exp Transl Clin. 2020 Dec 9;6(4):2055217320978511. doi: 10.1177/2055217320978511 33343920 PMC7731718

[25] Huff WX, Bam M, Shireman JM, Kwon JH, Song L, Newman S, et al. Aging- and Tumor-Mediated Increase in CD8+CD28- T Cells Might Impose a Strong Barrier to Success of Immunotherapy in Glioblastoma. Immunohorizons. 2021 Jun 8;5(6):395–409. doi: 10.4049/immunohorizons.2100008 34103370 PMC8591704

[26] Ebrahimkhani S, Vafaee F, Young PE, Hur SSJ, Hawke S, Devenney E, et al. Exosomal microRNA signatures in multiple sclerosis reflect disease status. Sci Rep. 2017 Oct 30;7(1):14293. doi: 10.1038/s41598-017-14301-3 29084979 PMC5662562

[27] Ebrahimkhani S, Vafaee F, Hallal S, Wei H, Lee MYT, Young PE, et al. Deep sequencing of circulating exosomal microRNA allows non-invasive glioblastoma diagnosis. NPJ Precis Oncol. 2018 Dec 12;2:28. doi: 10.1038/s41698-018-0071-0 30564636 PMC6290767

[28] Krämer A, Green J, Pollard J Jr, Tugendreich S. Causal analysis approaches in Ingenuity Pathway Analysis. Bioinformatics. 2014 Feb 15;30(4):523–30. doi: 10.1093/bioinformatics/btt703 24336805 PMC3928520

[29] Custodia A, Romaus-Sanjurjo D, Aramburu-Núñez M, Álvarez-Rafael D, Vázquez-Vázquez L, Camino-Castiñeiras J, et al. Ceramide/Sphingosine 1-Phosphate Axis as a Key Target for Diagnosis and Treatment in Alzheimer’s Disease and Other Neurodegenerative Diseases. Int J Mol Sci. 2022 Jul 22;23(15):8082. doi: 10.3390/ijms23158082 35897658 PMC9331765

[30] Kappos L, Antel J, Comi G, Montalban X, O’Connor P, Polman CH, et al. Oral fingolimod (FTY720) for relapsing multiple sclerosis. N Engl J Med. 2006 Sep 14;355(11):1124–40. doi: 10.1056/NEJMoa052643 16971719

[31] Lee KD, Chow WN, Sato-Bigbee C, Graf MR, Graham RS, Colello RJ, et al. FTY720 reduces inflammation and promotes functional recovery after spinal cord injury. J Neurotrauma. 2009 Dec;26(12):2335–44. doi: 10.1089/neu.2008.0840 19624262 PMC2850297

[32] Gao C, Qian Y, Huang J, Wang D, Su W, Wang P, et al. Three-Day Consecutive Fingolimod Administration Improves Neurological Functions and Modulates Multiple Immune Responses of CCI Mice. Mol Neurobiol. 2017 Dec;54(10):8348–8360. doi: 10.1007/s12035-016-0318-0 27924525

[33] Spampinato SF, Obermeier B, Cotleur A, Love A, Takeshita Y, Sano Y, et al. Sphingosine 1 Phosphate at the Blood Brain Barrier: Can the Modulation of S1P Receptor 1 Influence the Response of Endothelial Cells and Astrocytes to Inflammatory Stimuli? PLoS One. 2015 Jul 21;10(7):e0133392. doi: 10.1371/journal.pone.0133392 26197437 PMC4511229

[34] Johnson HL, Willenbring RC, Jin F, Manhart WA, LaFrance SJ, Pirko I, et al. Perforin competent CD8 T cells are sufficient to cause immune-mediated blood-brain barrier disruption. PLoS One. 2014 Oct 22;9(10):e111401. doi: 10.1371/journal.pone.0111401 25337791 PMC4206459

[35] Oukka M, Bettelli E. Regulation of lymphocyte trafficking in central nervous system autoimmunity. Curr Opin Immunol. 2018 Dec;55:38–43. doi: 10.1016/j.coi.2018.09.008 30268837 PMC6286213

[36] Hawke S, Zinger A, Juillard PG, Holdaway K, Byrne SN, Grau GE. Selective modulation of trans-endothelial migration of lymphocyte subsets in multiple sclerosis patients under fingolimod treatment. J Neuroimmunol. 2020 Dec 15;349:577392. doi: 10.1016/j.jneuroim.2020.577392 33007647

[37] Pournajaf S, Dargahi L, Javan M, Pourgholami MH. Molecular Pharmacology and Novel Potential Therapeutic Applications of Fingolimod. Front Pharmacol. 2022 Feb 16;13:807639. doi: 10.3389/fphar.2022.807639 35250559 PMC8889014

[38] Brinkmann V, Davis MD, Heise CE, Albert R, Cottens S, Hof R, et al. The immune modulator FTY720 targets sphingosine 1-phosphate receptors. J Biol Chem. 2002 Jun 14;277(24):21453–7. doi: 10.1074/jbc.C200176200 11967257

[39] Gräler MH, Goetzl EJ. The immunosuppressant FTY720 down-regulates sphingosine 1-phosphate G-protein-coupled receptors. FASEB J. 2004 Mar;18(3):551–3. doi: 10.1096/fj.03-0910fje 14715694

[40] Choi JW, Gardell SE, Herr DR, Rivera R, Lee CW, Noguchi K, et al. FTY720 (fingolimod) efficacy in an animal model of multiple sclerosis requires astrocyte sphingosine 1-phosphate receptor 1 (S1P1) modulation. Proc Natl Acad Sci U S A. 2011 Jan 11;108(2):751–6. doi: 10.1073/pnas.1014154108 21177428 PMC3021041

[41] Kim S, Bielawski J, Yang H, Kong Y, Zhou B, Li J. Functional antagonism of sphingosine-1-phosphate receptor 1 prevents cuprizone-induced demyelination. Glia. 2018 Mar;66(3):654–669. doi: 10.1002/glia.23272 29193293 PMC5773114

[42] Tonelli F, Lim KG, Loveridge C, Long J, Pitson SM, Tigyi G, et al. FTY720 and (S)-FTY720 vinylphosphonate inhibit sphingosine kinase 1 and promote its proteasomal degradation in human pulmonary artery smooth muscle, breast cancer and androgen-independent prostate cancer cells. Cell Signal. 2010 Oct;22(10):1536–42. doi: 10.1016/j.cellsig.2010.05.022 20570726 PMC2947314

[43] Kataoka H, Sugahara K, Shimano K, Teshima K, Koyama M, Fukunari A, et al. FTY720, sphingosine 1-phosphate receptor modulator, ameliorates experimental autoimmune encephalomyelitis by inhibition of T cell infiltration. Cell Mol Immunol. 2005 Dec;2(6):439–48. 16426494

[44] Foster CA, Mechtcheriakova D, Storch MK, Balatoni B, Howard LM, Bornancin F, et al. FTY720 rescue therapy in the dark agouti rat model of experimental autoimmune encephalomyelitis: expression of central nervous system genes and reversal of blood-brain-barrier damage. Brain Pathol. 2009 Apr;19(2):254–66. doi: 10.1111/j.1750-3639.2008.00182.x 18540945 PMC8094834

[45] Samuvel DJ, Saxena N, Dhindsa JS, Singh AK, Gill GS, Grobelny DW, et al. AKP-11—A Novel S1P1 Agonist with Favorable Safety Profile Attenuates Experimental Autoimmune Encephalomyelitis in Rat Model of Multiple Sclerosis. PLoS One. 2015 Oct 29;10(10):e0141781. doi: 10.1371/journal.pone.0141781 26513477 PMC4626178

[46] Bonfiglio T, Olivero G, Merega E, Di Prisco S, Padolecchia C, Grilli M, et al. Prophylactic versus Therapeutic Fingolimod: Restoration of Presynaptic Defects in Mice Suffering from Experimental Autoimmune Encephalomyelitis. PLoS One. 2017 Jan 26;12(1):e0170825. doi: 10.1371/journal.pone.0170825 28125677 PMC5268435

[47] Bonfiglio T, Olivero G, Merega E, Di Prisco S, Padolecchia C, Grilli M, et al. Correction: Prophylactic versus Therapeutic Fingolimod: Restoration of Presynaptic Defects in Mice Suffering from Experimental Autoimmune Encephalomyelitis. PLoS One. 2023 Oct 3;18(10):e0292584. doi: 10.1371/journal.pone.0292584 37788283 PMC10547186

[48] Nishihara H, Shimizu F, Sano Y, Takeshita Y, Maeda T, Abe M, et al. Fingolimod prevents blood-brain barrier disruption induced by the sera from patients with multiple sclerosis. PLoS One. 2015 Mar 16;10(3):e0121488. doi: 10.1371/journal.pone.0121488 25774903 PMC4361641

[49] Kano M, Kobayashi T, Date M, Tennichi M, Hamaguchi Y, Strasser DS, et al. Attenuation of murine sclerodermatous models by the selective S1P1 receptor modulator cenerimod. Sci Rep. 2019 Jan 24;9(1):658. doi: 10.1038/s41598-018-37074-9 30679645 PMC6345830

[50] Piali L, Froidevaux S, Hess P, Nayler O, Bolli MH, Schlosser E, et al. The selective sphingosine 1-phosphate receptor 1 agonist ponesimod protects against lymphocyte-mediated tissue inflammation. J Pharmacol Exp Ther. 2011 May;337(2):547–56. doi: 10.1124/jpet.110.176487 21345969

[51] Becker KA, Halmer R, Davies L, Henry BD, Ziobro-Henry R, Decker Y, et al. Blockade of Experimental Multiple Sclerosis by Inhibition of the Acid Sphingomyelinase/Ceramide System. Neurosignals. 2017;25(1):88–97. doi: 10.1159/000484621 29131010

[52] Lee EC, Lee YS, Park N, So KS, Chun YJ, Kim MY. Ceramide induces apoptosis and growth arrest of human glioblastoma cells by inhibiting Akt signaling pathways. Biomol Ther 2011;19:21–6. doi: 10.4062/biomolther.2011.19.1.021

[53] Doan NB, Alhajala H, Al-Gizawiy MM, Mueller WM, Rand SD, Connelly JM, et al. Acid ceramidase and its inhibitors: a de novo drug target and a new class of drugs for killing glioblastoma cancer stem cells with high efficiency. Oncotarget. 2017 Nov 7;8(68):112662–112674. doi: 10.18632/oncotarget.22637 29348854 PMC5762539

[54] Chang CY, Li JR, Wu CC, Wang JD, Yang CP, Chen WY, et al. Indomethacin induced glioma apoptosis involving ceramide signals. Exp Cell Res. 2018 Apr 1;365(1):66–77. doi: 10.1016/j.yexcr.2018.02.019 29470962

[55] Abuhusain HJ, Matin A, Qiao Q, Shen H, Kain N, Day BW, et al. A metabolic shift favoring sphingosine 1-phosphate at the expense of ceramide controls glioblastoma angiogenesis. J Biol Chem. 2013 Dec 27;288(52):37355–64. doi: 10.1074/jbc.M113.494740 24265321 PMC3873587

[56] Bien-Möller S, Lange S, Holm T, Böhm A, Paland H, Küpper J, et al. Expression of S1P metabolizing enzymes and receptors correlate with survival time and regulate cell migration in glioblastoma multiforme. Oncotarget. 2016 Mar 15;7(11):13031–46. doi: 10.18632/oncotarget.7366 26887055 PMC4914339

[57] Abdel Hadi L, Anelli V, Guarnaccia L, Navone S, Beretta M, Moccia F, et al. A bidirectional crosstalk between glioblastoma and brain endothelial cells potentiates the angiogenic and proliferative signaling of sphingosine-1-phosphate in the glioblastoma microenvironment. Biochim Biophys Acta Mol Cell Biol Lipids. 2018 Oct;1863(10):1179–1192. doi: 10.1016/j.bbalip.2018.07.009 30056170

[58] Chongsathidkiet P, Jackson C, Koyama S, Loebel F, Cui X, Farber SH, et al. Sequestration of T cells in bone marrow in the setting of glioblastoma and other intracranial tumors. Nat Med. 2018 Sep;24(9):1459–1468. doi: 10.1038/s41591-018-0135-2 30104766 PMC6129206

[59] Chongsathidkiet P, Jackson C, Koyama S, Loebel F, Cui X, Farber SH, et al. Author Correction: Sequestration of T cells in bone marrow in the setting of glioblastoma and other intracranial tumors. Nat Med. 2019 Mar;25(3):529. doi: 10.1038/s41591-019-0355-0 30670876 PMC6825406

[60] Onishi H, Kiyota A, Koya N, Tanaka H, Umebayashi M, Katano M, et al. Random migration contributes to cytotoxicity of activated CD8+ T-lymphocytes but not NK cells. Anticancer Res. 2014 Aug;34(8):3947–56. 25075016

[61] Xiong Y, Piao W, Brinkman CC, Li L, Kulinski JM, Olivera A, et al. CD4 T cell sphingosine 1-phosphate receptor (S1PR)1 and S1PR4 and endothelial S1PR2 regulate afferent lymphatic migration. Sci Immunol. 2019 Mar 15;4(33):eaav1263. doi: 10.1126/sciimmunol.aav1263 30877143 PMC6744614

[62] Drouillard A, Neyra A, Mathieu AL, Marçais A, Wencker M, Marvel J, et al. Human Naive and Memory T Cells Display Opposite Migratory Responses to Sphingosine-1 Phosphate. J Immunol. 2018 Jan 15;200(2):551–557. doi: 10.4049/jimmunol.1701278 29237776

[63] Olesch C, Sirait-Fischer E, Berkefeld M, Fink AF, Susen RM, Ritter B, et al. S1PR4 ablation reduces tumor growth and improves chemotherapy via CD8+ T cell expansion. J Clin Invest. 2020 Oct 1;130(10):5461–5476. doi: 10.1172/JCI136928 32663191 PMC7524469

[64] Burkard T, Dreis C, Herrero San Juan M, Huhn M, Weigert A, Pfeilschifter JM, et al. Enhanced CXCR4 Expression of Human CD8Low T Lymphocytes Is Driven by S1P4. Front Immunol. 2021 Aug 24;12:668884. doi: 10.3389/fimmu.2021.668884 34504486 PMC8421764

[65] Heinrich M, Neumeyer J, Jakob M, Hallas C, Tchikov V, Winoto-Morbach S, et al. Cathepsin D links TNF-induced acid sphingomyelinase to Bid-mediated caspase-9 and -3 activation. Cell Death Differ. 2004 May;11(5):550–63. doi: 10.1038/sj.cdd.4401382 14739942

[66] Appelqvist H, Johansson AC, Linderoth E, Johansson U, Antonsson B, Steinfeld R, et al. Lysosome-mediated apoptosis is associated with cathepsin D-specific processing of bid at Phe24, Trp48, and Phe183. Ann Clin Lab Sci. 2012 Summer;42(3):231–42. 22964611

[67] Lin CF, Tsai CC, Huang WC, Wang YC, Tseng PC, Tsai TT, et al. Glycogen Synthase Kinase-3β and Caspase-2 Mediate Ceramide- and Etoposide-Induced Apoptosis by Regulating the Lysosomal-Mitochondrial Axis. PLoS One. 2016 Jan 4;11(1):e0145460. doi: 10.1371/journal.pone.0145460 26727221 PMC4699703

[68] Wu J, Huang Y, Xie Q, Zhang J, Zhan Z. A novel bis-aryl urea compound inhibits tumor proliferation via cathepsin D-associated apoptosis. Anticancer Drugs. 2020 Jun;31(5):500–506. doi: 10.1097/CAD.0000000000000898 31917700 PMC7147394

[69] Seo SU, Woo SM, Im SS, Jang Y, Han E, Kim SH, et al. Cathepsin D as a potential therapeutic target to enhance anticancer drug-induced apoptosis via RNF183-mediated destabilization of Bcl-xL in cancer cells. Cell Death Dis. 2022 Feb 4;13(2):115. doi: 10.1038/s41419-022-04581-7 35121737 PMC8816936

[70] Heinrich M, Wickel M, Schneider-Brachert W, Sandberg C, Gahr J, Schwandner R, et al. Cathepsin D targeted by acid sphingomyelinase-derived ceramide. EMBO J. 1999 Oct 1;18(19):5252–63. doi: 10.1093/emboj/18.19.5252 10508159 PMC1171596

[71] Brunner J, Kronke M, Schutze S, Baumann M, Feederle R, Kremmer E, et al. Cathepsin D targeted by acid sphingomyelinase-derived ceramide M. Heinrich, M. Wickel, W. Schneider-Brachert, C. Sandberg, J. Gahr, R. Schwandner, et al. The EMBO Journal. 2000;19(2):315. doi: 10.1093/emboj/190315a PMC1171596 10508159

[72] Prieto A, Díaz D, Barcenilla H, Castrillo C, Monserrat J, Merino AG, et al. Increased spontaneous ex vivo apoptosis and subset alterations in peripheral blood T cells from patients with multiple sclerosis. J Clin Immunol. 2006 Mar;26(2):101–12. doi: 10.1007/s10875-006-9007-5 16758338

[73] Grecchi S, Mazzini G, Lisa A, Armentero MT, Bergamaschi R, Romani A, et al. Search for cellular stress biomarkers in lymphocytes from patients with multiple sclerosis: a pilot study. PLoS One. 2012;7(9):e44935. doi: 10.1371/journal.pone.0044935 23028690 PMC3441649

[74] Grecchi S, Mazzini G, Lisa A, Armentero M-T, Bergamaschi R, Romani A, et al. (2012) Correction: Search for Cellular Stress Biomarkers in Lymphocytes from Patients with Multiple Sclerosis: A Pilot Study. PLoS One. 2012;7(11): doi: 10.1371/annotation/8c8710a2-bd43-4f65-b21e-69ec522c4f22 PMC3441649 23028690

[75] Morford LA, Dix AR, Brooks WH, Roszman TL. Apoptotic elimination of peripheral T lymphocytes in patients with primary intracranial tumors. J Neurosurg. 1999 Dec;91(6):935–46. doi: 10.3171/jns.1999.91.6.0935 10584838

[76] Lamano JB, Lamano JB, Li YD, DiDomenico JD, Choy W, Veliceasa D, et al. Glioblastoma-Derived IL6 Induces Immunosuppressive Peripheral Myeloid Cell PD-L1 and Promotes Tumor Growth. Clin Cancer Res. 2019 Jun 15;25(12):3643–3657. doi: 10.1158/1078-0432.CCR-18-2402 30824583 PMC6571046

[77] Marx S, Splittstöhser M, Kinnen F, Moritz E, Joseph C, Paul S, et al. Platelet activation parameters and platelet-leucocyte-conjugate formation in glioblastoma multiforme patients. Oncotarget. 2018 May 25;9(40):25860–25876. doi: 10.18632/oncotarget.25395 29899827 PMC5995223

[78] Kułakowska A, Zendzian-Piotrowska M, Baranowski M, Konończuk T, Drozdowski W, Górski J, et al. Intrathecal increase of sphingosine 1-phosphate at early stage multiple sclerosis. Neurosci Lett. 2010 Jun 25;477(3):149–52. doi: 10.1016/j.neulet.2010.04.052 20434523

[79] Filippatou AG, Moniruzzaman M, Sotirchos ES, Fitzgerald KC, Kalaitzidis G, Lambe J, et al. Serum ceramide levels are altered in multiple sclerosis. Mult Scler. 2021 Sep;27(10):1506–1519. doi: 10.1177/1352458520971816 33307993 PMC8200368

[80] Ebrahimkhani S, Beadnall HN, Wang C, Suter CM, Barnett MH, Buckland ME, et al. Serum Exosome MicroRNAs Predict Multiple Sclerosis Disease Activity after Fingolimod Treatment. Mol Neurobiol. 2020 Feb;57(2):1245–1258. doi: 10.1007/s12035-019-01792-6 31721043

[81] Vignard V, Labbé M, Marec N, André-Grégoire G, Jouand N, Fonteneau JF, et al. MicroRNAs in Tumor Exosomes Drive Immune Escape in Melanoma. Cancer Immunol Res. 2020 Feb;8(2):255–267. doi: 10.1158/2326-6066.CIR-19-0522 31857348

[82] Liu ZM, Wang YB, Yuan XH. Exosomes from murine-derived GL26 cells promote glioblastoma tumor growth by reducing number and function of CD8+T cells. Asian Pac J Cancer Prev. 2013;14(1):309–14. doi: 10.7314/apjcp.2013.14.1.309 23534743

[83] Azambuja JH, Ludwig N, Yerneni S, Rao A, Braganhol E, Whiteside TL. Molecular profiles and immunomodulatory activities of glioblastoma-derived exosomes. Neurooncol Adv. 2020 May 6;2(1):vdaa056. doi: 10.1093/noajnl/vdaa056 32642708 PMC7262743

[84] Guo X, Qiu W, Wang J, Liu Q, Qian M, Wang S, et al. Glioma exosomes mediate the expansion and function of myeloid-derived suppressor cells through microRNA-29a/Hbp1 and microRNA-92a/Prkar1a pathways. Int J Cancer. 2019 Jun 15;144(12):3111–3126. doi: 10.1002/ijc.32052 30536597

[85] Qiu W, Guo X, Li B, Wang J, Qi Y, Chen Z, et al. Exosomal miR-1246 from glioma patient body fluids drives the differentiation and activation of myeloid-derived suppressor cells. Mol Ther. 2021 Dec 1;29(12):3449–3464. doi: 10.1016/j.ymthe.2021.06.023 34217892 PMC8636176

[86] Soheilifar MH, Vaseghi H, Seif F, Ariana M, Ghorbanifar S, Habibi N, et al. Concomitant overexpression of mir-182-5p and mir-182-3p raises the possibility of IL-17-producing Treg formation in breast cancer by targeting CD3d, ITK, FOXO1, and NFATs: A meta-analysis and experimental study. Cancer Sci. 2021 Feb;112(2):589–603. doi: 10.1111/cas.14764 33283362 PMC7893989

[87] Kundu ST, Rodriguez BL, Gibson LA, Warner AN, Perez MG, Bajaj R, et al. The microRNA-183/96/182 cluster inhibits lung cancer progression and metastasis by inducing an interleukin-2-mediated antitumor CD8+ cytotoxic T-cell response. Genes Dev. 2022 May 1;36(9–10):582–600. doi: 10.1101/gad.349321.121 35654454 PMC9186390

[88] Li J, Yuan H, Xu H, Zhao H, Xiong N. Hypoxic Cancer-Secreted Exosomal miR-182-5p Promotes Glioblastoma Angiogenesis by Targeting Kruppel-like Factor 2 and 4. Mol Cancer Res. 2020 Aug;18(8):1218–1231. doi: 10.1158/1541-7786.MCR-19-0725 32366676

[89] Schneider B, William D, Lamp N, Zimpfer A, Henker C, Classen CF, et al. The miR-183/96/182 cluster is upregulated in glioblastoma carrying EGFR amplification. Mol Cell Biochem. 2022 Sep;477(9):2297–2307. doi: 10.1007/s11010-022-04435-y 35486213 PMC9395473

[90] Kouri FM, Hurley LA, Daniel WL, Day ES, Hua Y, Hao L, et al. miR-182 integrates apoptosis, growth, and differentiation programs in glioblastoma. Genes Dev. 2015 Apr 1;29(7):732–45. doi: 10.1101/gad.257394.114 25838542 PMC4387715

